# Impact of Filgrastim on Mortality During Induction Chemotherapy in Childhood B-Cell Non-Hodgkin Lymphoma

**DOI:** 10.7759/cureus.77320

**Published:** 2025-01-12

**Authors:** Madiha Jameel, Rabia Wali, Syed Muhammad Jawad Zaidi, Najma Shaheen, Irfana Ishaq Sindhu

**Affiliations:** 1 Pediatric Oncology, Shaukat Khanum Memorial Cancer Hospital and Research Centre, Lahore, PAK

**Keywords:** b-cell non-hodgkin lymphoma, febrile neutropenia, filgrastim, induction chemotherapy, mortality

## Abstract

Background

Childhood B-cell non-Hodgkin lymphoma (NHL) is a group of rapidly growing neoplasms that are fatal if left untreated. Induction chemotherapy during the treatment makes these patients vulnerable to several life-threatening infections due to their immunocompromised state. Prophylactic use of filgrastim before initiating the second induction chemotherapy cycle can improve outcomes. Therefore, our study aims to assess various parameters that can lead to acute mortality during induction chemotherapy and how the prophylactic use of filgrastim can prevent it.

Methods

This retrospective study was performed at Shaukat Khanum Memorial Cancer Hospital and Research Centre, Lahore, Pakistan. All patients of B-cell NHL under 10 years of age diagnosed between January 2018 and October 2021 were included. Various parameters including patient age, stage of disease, treatment regimen, day of the start of filgrastim, number of episodes of febrile neutropenia, duration of admission in hospital due to febrile neutropenia, day of count recovery, and outcomes were collected.

Results

Of the 106 patients, 45 (42.4%) were female and 61 (57.5%) were male; 60 patients (56.7%) were between one and five years of age. All patients were started on granulocyte colony-stimulating factor (G-CSF) on the seventh day of the first induction chemotherapy cycle. A total of 97 patients (91.5%) remained alive until the end of induction, and nine patients (8.5%) succumbed due to febrile neutropenia complications. The median days for filgrastim were eight (0-20) days after completion of the first cycle of COPADM (cyclophosphamide, vincristine, prednisolone, doxorubicin, and methotrexate). On applying the Mann-Whitney U test, there was a significant difference in the day of count recovery among infected and non-infected patients (p = 0.01).

Conclusions

Primary prophylactic use of filgrastim can significantly reduce mortality during induction chemotherapy in childhood B-cell NHL. Patients had lower febrile neutropenia episodes, decreased length of hospital stay, and, early count recovery. Therefore, filgrastim before the second cycle of induction chemotherapy, especially in resource-limited settings, can yield better outcomes.

## Introduction

Non-Hodgkin lymphoma (NHL) ranks as the fifth most prevalent cancer among children under 15 years old [1]. Among malignant lymphomas, mature B-cell NHL is the most common type, representing approximately 7% of all childhood cancers [2]. These malignancies are notably aggressive and have high mortality without treatment, however, with timely treatment they respond extremely well [3]. The treatment comes with its challenges, including severe hematological toxicity, mucositis, bone marrow suppression, heightened risk of severe infection due to febrile neutropenia, and extended hospital stays [2,4]. Through a risk-stratified approach, intensified chemotherapy, incorporation of immunotherapy, and vigorous supportive measures, high-income countries have achieved improved outcomes, with cure rates ranging from 85% to 95% across all risk categories [5]. Unfortunately, in low to middle-income countries, cure rates still fluctuate between 20% and 70%, primarily due to inadequate diagnostic capabilities, delayed diagnoses, treatment abandonment, toxicities leading to death, malnutrition, tumor lysis syndrome (TLS), sepsis, and relapse [5,6].

Literature from Pakistan reports an acute mortality rate of 19.7%, identifying several contributing factors to the high incidence of early deaths in NHL cases [2]. Studies from developing countries report tumor lysis syndrome, severe neutropenic sepsis, and malnutrition to be the main culprits of mortality [2,6]. While the utilization of G-CSF or filgrastim has shown efficacy in reducing the duration and severity of neutropenia, there is a notable lack of research specifically evaluating its outcomes in resource-limited settings [7]. Most existing studies originate from high-income countries, which may not reflect the unique challenges faced in low-resource environments, such as delayed access to healthcare, inconsistent availability of medications, and higher malnutrition and infection baseline rates. Exploring these gaps could provide valuable insights into optimizing the use of filgrastim and improving outcomes in these vulnerable populations.

This study aims to explore the impact of G-CSF on mortality when used as a primary prophylaxis during induction chemotherapy in B-cell NHL cases. Our study also aims to assess its effect on hospitalization days, febrile neutropenia episodes, and preventing delays in commencing chemotherapy cycles.

This article was previously posted on the preprint server, Authorea, on June 2, 2024 [8].

## Materials and methods

This was a retrospective study conducted at the Shaukat Khanum Memorial Cancer Hospital, Lahore, Pakistan. All children diagnosed with pediatric B-cell NHL, stage III and IV, under 10 years of age from January 2018 to October 2021, were enrolled in the study. The study was approved by the Institutional Review Board of Shaukat Khanum Memorial Cancer Hospital (exemption number: EX-03-03-22-01-A2). Patients were identified through consecutive admissions to the Pediatric Oncology Department at the Shaukat Khanum Memorial Cancer Hospital. A retrospective review of medical records ensured comprehensive data collection. Potential participants who did not complete the induction chemotherapy protocol or had missing critical data were excluded from the analysis. The reasons for exclusion, such as treatment abandonment or transfer to other facilities, were documented. This age limit of 10 years was due to the institutional acceptance policy.

The diagnosis was confirmed on morphology and immunohistochemistry. Staging was conducted following the St. Jude Children's Research Hospital staging system [9], utilizing computed tomography (CT) scans. Central nervous system (CNS) involvement was defined as the presence of blast cells in the cerebrospinal fluid, cranial nerve palsy, clinical evidence of spinal cord compression, isolated intracerebral mass, or para-meningeal extension involving the cranial or spinal regions. A bone marrow aspiration containing more than 25% blast cells was considered positive. Stages I and II were categorized as Low, while Stages III and IV were classified as Advanced. Patients were further stratified into treatment groups A, B, and C based on staging and resection status, following the guidelines outlined in the United Kingdom Children's Cancer Study Group (UKCCSG) 2003 recommendations [2]. These details are shown in Table 1. 

**Table 1:** Tabulated Description of staging and treatment groups.

Treatment Group	Description of Disease
Group A	Completely resected stage I
	Completely resected abdominal stage II
Group B	Unresected stage I and II disease
	Resected stage II disease other than abdominal completely resected tumors
	Stage III disease and bone marrow involvement more than 25%
Group C	Any Central nervous system involvement or bone marrow involvement more than 25%

Initial cytoreductive chemotherapy comprised cyclophosphamide, vincristine, and prednisolone (COP), supplemented with intrathecal chemotherapy involving methotrexate and hydrocortisone. All chemotherapy drugs including the intrathecal chemotherapy were given on day one followed by steroids for six more days. For clinically unstable patients, a second course of COP was administered.

Response to treatment scans were performed on day seven of COP administration. The treatment regimen was escalated from group B to C if there was less than a 20% response. Subsequently, the first cycle of induction chemotherapy with vincristine (1.5 mg/m² intravenously (IV), capped at a maximum dose of 2 mg), prednisolone (60 mg/m² orally or IV, daily on days 1-5), methotrexate, cyclophosphamide (250 mg/m² IV, daily on days 1-5), and doxorubicin (60 mg/m² IV on day 1). This is called the COPADM regimen. Intrathecal chemotherapies with methotrexate, hydrocortisone, and cytarabine were also part of the regimen. The dose for methotrexate was 3 g/m^2^ in patients in treatment group B and 8 g/m^2^ in treatment group C, along with more intensive CNS-based chemotherapy with high dose cytarabine. Details of treatment plans in treatment groups are shown in Table 2.

**Table 2:** Details of treatment regimens in different treatment groups. COP: cyclophosphamide, vincristine, and prednisolone; COPAD: cyclophosphamide, vincristine, prednisolone, and doxorubicin; COPADM: cyclophosphamide, vincristine, prednisolone, doxorubicin, and methotrexate; CYM: cytarabine and methotrexate; CYVE: high-dose cytarabine and etoposide

Treatment Group	Cytoreductive chemotherapy regimen	Induction chemotherapy regimen	Consolidation chemotherapy regimen	Maintenance chemotherapy regimen
Group A	Not required	2 cycles of COPAD	Not required	Not required
Group B	1-2 cycles of COP	2 cycles of COPADM	2 cycles of CYM	Not required
Group C	1-2 cycles of COP	2 cycles of COPADM	2 cycles of CYVE	1-4 Maintenance Cycles

The 2003 guidelines did not specifically recommend using prophylactic filgrastim; however, because of high acute mortality based on our own published results [2], our institutional guideline recommended giving primary prophylaxis with filgrastim at the dose of 5 mcg/kg subcutaneously, 24 hours after the last dose of chemotherapy. Two cycles of COPADM were spaced 21 days apart upon count recovery. G-CSF was initiated on day 7 of COPADM for all patients and blood counts were monitored for recovery and neutropenic episodes.

Febrile neutropenia was defined as a core body temperature of more than 38.2°C or ≥ 38.0°C for more than one hour along with absolute neutrophil count (ANC) of less than 1000/μL. The presence of positive blood cultures or clinical criteria, if cultures were negative, was labeled as a febrile neutropenia episode. Probable fungal infection was diagnosed and treated based on radiographic or histological findings. Count recovery was defined as an ANC count of more than 1000/μL and a platelets count of more than 100/μL. The duration of a febrile neutropenic episode was measured from the onset of fever until the patient was afebrile for 48 hours.

Acute mortality was defined as death before the administration of the second induction course of COPADM. Patients presenting with fever were admitted, and data were collected on the duration of stay, febrile neutropenic episodes, acute mortality rate, days of count recovery, delay in chemotherapy, and cause of death. Once the patient was clinically stable and counts had recovered, the second cycle of COPADM was administered. Data on patient age, stage of disease, treatment regimen, day of start of filgrastim, number of episodes of febrile neutropenia, duration of admission in hospital due to febrile neutropenia, day of count recovery, and disease outcomes.

Categorial variables were expressed as frequencies and percentages while numerical variables were expressed with means and standard deviation along with medians and interquartile ranges. Mann-Whitney U test was performed to assess the difference in median days of count recovery and hospital stay in infected versus non-infected patients. A p-value of less than 0.05 was considered statistically significant. IBM SPSS Statistics for Windows, Version 25.0 (Released 2017; IBM Corp., Armonk, New York, United States) was utilized to analyze data.

## Results

Of the total of 106 patients, 45 (42.4%) were female and 61 (57.5%) were male. As far as age was concerned, 60 (56.7%) were in the age group of one to five years while the remaining 46 (43.3%) were between 6-10 years of age. Eighty-one (76.4%) patients had stage III disease while 25 (23.6%) had stage IV disease. The details of clinical characteristics are shown in Table 3.

**Table 3:** Details of clinical parameters of the study participants.

Parameters		Frequency	Percentages
Gender	Male	61	56.7%
	Female	45	42.4%
Disease Stage	Stage III	81	76.4%
	Stage IV	25	23.6%
Age Group	1-5 years	60	56.7%
	6-10 years	46	43.3%
Treatment Regimen	Group B	79	74.5%
	Group C	25	25.5%
Type of Non-Hodgkin Lymphoma	Abdominal Burkitt’s lymphoma	99	92.4%
	Abdominal Diffuse Large B-cell lymphoma	4	3.77%
	Oropharyngeal Burkitt’s Lymphoma	1	0.94%
	Paraspinal Burkitt’s Lymphoma	1	0.94%
	Left Orbital Burkitt’s Lymphoma	1	0.94%

In all patients, filgrastim was started on day 7 of the first cycle of COPADM. The second cycle of induction chemotherapy was given on the 21st day in 57 (53.8%) on count recovery; it was delayed in the remaining patients due to prolonged febrile neutropenia. A total of 97 patients (91.5%) remained alive till the end of induction and nine patients expired with a mortality rate of 8.5%. A total of 48 (45.4%) patients were discharged on the seventh day after the first cycle, 41 (38.6%) remained admitted after completion of chemotherapy for 8-15 days due to febrile neutropenia, and 17 (16%) patients had inpatient stay of more than 15 days due to prolonged febrile neutropenia. The details are shown in Table 4.

**Table 4:** Details of patient outcomes. FN: febrile neutropenia

Parameters		Frequency	Percentages
Mortality status	Dead	9	8.5%
	Alive	97	91.5%
Febrile neutropenia episodes	Single	102	96.2%
	Multiple	4	3.8%
Bacterial infection	Yes	34	32%
	No	72	68%
Fungal Infection	Yes	10	9.5%
	No	96	90.5%
Duration of admission in FN episode	1-7	48	45.3%
	8-15	41	38.7%
	More than 15	17	16%
Need for Intensive care unit	Yes	16	15.1%
	No	90	84.9%

Regarding the cause of infection, 34 patients had evidence of infection with a positive blood culture, with gram-negative rods being the most common isolates. Multi-drug resistant gram-negative/positive bacteremia and invasive fungal infections remain the most frequent causes of prolonged febrile neutropenia in these patients. Organisms included vancomycin-resistant *Enterococcus*, methicillin-sensitive *Staphylococcus aureus*, methicillin-resistant *S. aureus*, *Escherichia coli*, *Stenotrophomonas*, *Pseudomonas*, yeast, and aspergillus. This is delineated in Table 5.

**Table 5:** A delineation of organisms isolated from blood cultures.

Type of Isolate	Organism Isolated in blood culture	Frequency	Percentage
Gram Positive	Methicillin-sensitive *Staphylococcus aureus*	4	11.8%
	Methicillin-resistant *Staphylococcus aureus*	2	5.8%
	Vancomycin-sensitive *Enterococcus*	1	2.9%
	Vancomycin-resistant *Enterococcus*	2	5.8%
Gram Negative isolates	Escherichia coli	11	32.3%
	Acinetobacter	1	2.9%
	Klebsiella	1	3.9%
	Pseudomonas	4	11.8%
	Stenotrophomonas	3	8.8%
Fungal Isolates	Aspergillus fumigatus	2	5.9%
	Yeast cells/*Candida*	3	8.8%

Filgrastim was administered to the patients till count recovery. The median number of days for use of filgrastim was eight (0-20) days after completion of the first cycle of COPADM. On applying the Mann-Whitney U test, there was a significant difference in the day of count recovery among infected and non-infected patients (p = 0.01). The difference in medians of infected and non-infected patients concerning other parameters are shown in Table 6.

**Table 6:** Difference in median days of count recovery with various parameters in infected versus non-infected patients. COPADM: cyclophosphamide, vincristine, prednisolone, doxorubicin, and methotrexate

Parameter	Infected, median (IQR)	Non-Infected, median (IQR)	p-value
Day of count recovery	9.5 (0-20)	7 (1-16)	0.01
Day of the start of second cycle of COPADM	21 (0-42)	21 (0-27)	0.22
Day of discharge	9.5 (2-28)	8 (2-53)	0.61

## Discussion

Childhood NHL is characterized by high grade and aggressive nature [10]. Prognosis in developed countries has shown steady improvement, with survival rates reaching as high as 80-90% [11]. The most effective treatment strategies have emerged from three major childhood cancer groups, including the European lymphoma malignancy B-cell (LMB), Berlin Frankfurt-Munster (BFM), and Children Oncology Group (COG) [11,12]. Despite advancements, treatment-related toxicities such as hematological toxicity, prolonged mucositis, and susceptibility to serious infections remain significant concerns. However, enhancements in supportive care have contributed to improved outcomes. Studies from the developed world showed low mortality rates between 2-3% with no early deaths [12].

Outcomes for pediatric NHL are poor in low-income countries, where increased rates of early and toxic deaths lead to low survival rates. A study from our institution, by Raheela et al., reported an acute mortality rate of 19.7%, identifying several contributing factors to the high incidence of early deaths [2]. These included TLS, malnutrition, and neutropenic sepsis after the first cycle of induction chemotherapy. Of the patients, 42.9% developed severe neutropenic sepsis with bacterial culture-proven neutropenic sepsis in 71% while 19.7% developed fungal infections. After the results of the study by Raheela et al. [2], it was decided in 2018 as part of our departmental guidelines to give primary prophylaxis of filgrastim to all patients of B cell-NHL to improve the mortality and febrile neutropenic complications, contrary to the UKCCSG guidelines that do not recommend prophylactic use of filgrastim. In the current study, we focused more on the infectious complications of acute mortality and the use of filgrastim. We observed a reduction in mortality from 19.7% to 8.5% with filgrastim as primary prophylaxis. Similarly, there was a reduction in hospital admission days with febrile neutropenia and delays in chemotherapy cycles which is of significant impact in our settings. These findings are compared to the study conducted by Raheela et al. at Shaukat Khanum Hospital [2]. 

Another study conducted in Pakistan reported infection-related mortality to be approximately 28.4% during induction chemotherapy in children with B cell NHL [13]. Similarly, studies in developing countries reported mortality rates of 20-30% during induction chemotherapy [14,15]. These findings highlight the need for therapeutic adaptations tailored to local conditions in low-income countries to mitigate treatment-related mortality.

Filgrastim is used to enhance early count recovery in neutropenic patients with myelosuppression secondary to chemotherapy or radiation [15]. Neutropenia, a common side effect of cancer chemotherapy, often leads to infections, with febrile neutropenia affecting a significant number of patients [4]. Febrile neutropenia frequently necessitates chemotherapy delays or dose modifications, prolongs hospital stay, increases costs, and diminishes patient quality of life. Overall survival benefit of 3.2% (95%CI: 2.1-4.2%) has been reported from its use with intensive chemotherapy [16]. While clinical trials have not significantly impacted overall or disease-free survival, they have demonstrated the importance of maintaining chemotherapy schedules, especially in curative settings [15,16].

The American Society of Clinical Oncology (ASCO) recommends that the prophylactic use of filgrastim helps to reduce the risk of febrile neutropenia, particularly when the risk is approximately 20% or higher [17]. Primary prophylaxis is recommended for reducing febrile neutropenic episodes and severity in patients at high risk based on age, medical history, disease characteristics, and the myelotoxicity associated with the chemotherapy regimen [17,18]. Meta-analysis on the use of filgrastim showed a 20% reduction in febrile neutropenia and shorter duration of hospitalization [19].

Moreover, it is evident in our study that there is a significant difference in days of count recovery between infected and non-infected patients. Filgrastim helps early count recovery and reduces febrile neutropenia-related complications. With early count recovery, we can prevent delays in planned chemotherapy and, hence, can have better outcomes. Furthermore, filgrastim helps prevent prolonged stay in hospital due to febrile neutropenia and, hence, reduces infection-related complications and toxic deaths. A study demonstrated that the use of filgrastim effectively enhances adherence to the planned treatment schedule, without impacting failure-free or overall survival rates. Moreover, its use was associated with a clinically significant reduction in the number of toxic deaths [20].

A retrospective study design and a single-center study account for our study limitations. The practice of using filgrastim as primary prophylaxis in different institutions across the country was not uniform at the time of this study duration. As a result, a prospective trial was initiated in 2022 and is currently undergoing on the national level in Pakistan to see if this intervention is statistically effective in the reduction of acute mortality (Prospective Study: Rabia Wali et al.. National Standard of Care Protocol for Treatment of Pediatric Mature B cell Non-Hodgkin Lymphoma (NBC-831). Approval Number: No.4-87/NBC-831/22/ 310, National Institutes of Health Health Research Institute National Bioethics Committee (NBC); September 7, 2022). This study will provide background for the future prospective study as there is very limited literature available locally.

The retrospective design of the current study provides valuable insights into real-world outcomes of filgrastim use in a resource-limited setting over four years. Despite variability in its administration, the significant reduction in mortality and febrile neutropenia episodes highlights its clinical efficacy. Differences in healthcare infrastructure and patient demographics at Shaukat Khanum Memorial Cancer Hospital compared to other settings show the importance and application of these findings. While in line with global literature, the study uniquely addresses challenges in low- and middle-income countries, such as multidrug-resistant infections and malnutrition. These results advocate for standardized filgrastim use and further research to optimize its cost-effectiveness and impact on patient outcomes.

## Conclusions

Primary prophylactic use of filgrastim significantly reduces mortality, improves count recovery, and decreases the number of febrile neutropenia episodes. All these factors prevent prolonged hospital stays and reduce delays at the start of the next chemotherapy cycle. Therefore, the use of filgrastim as primary prophylaxis after completion of the first cycle of induction chemotherapy needs to be promoted in pediatric cancer care centers, especially in resource-limited settings to improve survival outcomes. Further clinical trials at the national and international level are mandated to corroborate our findings.
